# Intravital imaging of fluorescent markers and FRET probes by DNA tattooing

**DOI:** 10.1186/1472-6750-7-2

**Published:** 2007-01-03

**Authors:** Adriaan D Bins, Jacco van Rheenen, Kees Jalink, Jonathan R Halstead, Nullin Divecha, David M Spencer, John BAG Haanen, Ton NM Schumacher

**Affiliations:** 1 Department of Immunology, The Netherlands Cancer Institute, Plesmanlaan 121, 1066 CX Amsterdam, The Netherlands; 2 Department of Anatomy and Structural Biology, Albert Einstein College of Medicine, 1300 Morris Park Avenue, Bronx, NY 10461, USA; 3 Department of Cell Biology, The Netherlands Cancer Institute, Plesmanlaan 121, 1066 CX Amsterdam, The Netherlands; 4 Department of Cellular Biochemistry, The Netherlands Cancer Institute, Plesmanlaan 121, 1066 CX Amsterdam, The Netherlands; 5 Department of Immunology, Baylor College of Medicine, Texas Medical Center, One Baylor Plaza, BCMM-M929, Houston, TX 77030, USA

## Abstract

**Background:**

Advances in fluorescence microscopy and mouse transgenesis have made it possible to image molecular events in living animals. However, the generation of transgenic mice is a lengthy process and intravital imaging requires specialized knowledge and equipment. Here, we report a rapid and undemanding intravital imaging method using generally available equipment.

**Results:**

By DNA tattooing we transfect keratinocytes of living mice with DNA encoding fluorescent biosensors. Subsequently, the behavior of individual cells expressing these biosensors can be visualized within hours and using conventional microscopy equipment. Using this "instant transgenic" model in combination with a corrected coordinate system, we followed the *in vivo *behavior of individual cells expressing either FRET- or location-based biosensors for several days. The utility of this approach was demonstrated by assessment of *in vivo *caspase-3 activation upon induction of apoptosis.

**Conclusion:**

This "instant skin transgenic" model can be used to follow the *in vivo *behavior of individual cells expressing either FRET- or location-based probes for several days after tattooing and provides a rapid and inexpensive method for intravital imaging in murine skin.

## Background

The introduction of fluorescent proteins (FP) and the ability to visualize molecular events using fluorescence microscopy have been of substantial value for understanding intracellular signaling events. FP-based biosensors, and especially those based on Fluorescent Resonance Energy Transfer (FRET), can report on cellular processes such as apoptosis or cell division, the levels of second messengers such as Ca^2+^, PtdIns(4,5)*P*_2 _and cAMP, or the activation status of proteins such as RhoA [1]. To date, the vast majority of analyses utilizing FP-based biosensors have been carried out on cells in tissue culture. However, for cellular processes that are controlled by cellular interactions or by the microenvironment, it would clearly be preferable to perform such analyses *in vivo*. Indeed, the small number of studies that have analyzed the behavior of single cells *in vivo*, have been informative with regard to the intracellular interactions of immune cells and early events in tumors formation [2-5]. Intravital images of whole mice or tumors can readily be acquired with macroscopical resolution using simple instrumentations (e.g. LED flashlight, filters and a digital camera [6]). However, for imaging with subcellular resolution, sophisticated equipment and expertise (e.g. two-photon microscopes [7] or whole-mouse imaging systems [8]) are required, which are only available in few specialized laboratories. Furthermore, the generation of biosensor-transgenic mice is a lengthy process and thereby hampers the use of this technique in settings where a number of different biosensors are tested or combinations of biosensors are required. A few studies have previously described fast and simple techniques to target selective genes to specific sites. For example, Li and Hoffman have targeted reporter genes to hair follicles in mice using liposomes [9]. However, it will be important to extend such approaches to other cell types. Here we present a rapid and inexpensive DNA tattoo method to target FP-genes to keratinocytes in the skin of mice. The behavior of these keratinocytes expressing fluorescent or FRET markers were followed for multiple days with subcellular resolution using a standard confocal microscope. The utility of this tattoo-approach was demonstrated by *in vivo *imaging of caspase-3 activation upon apoptosis induction.

## Results and Discussion

Prior experiments have demonstrated the feasibility of introducing transgenes in murine skin cells by use of a rapidly oscillating tattoo machine [10]. In an effort to determine the number of cells that express the introduced transgene upon this type of skin application (see Methods), we tattooed a small area (10 × 15 mm) of the abdominal skin of mice with DNA encoding a GFP reporter protein driven by the CMV immediate early (CMV-IE) promoter. 3 hours thereafter, mice were anesthetized and analyzed for DNA tattoo-induced GFP expression by intravital imaging using a conventional confocal microscope. Expression of the GFP reporter gene was observed in approximately 160 cells per 10 mm^2 ^tattooed skin, showing that the transfection efficiency of DNA tattooing was sufficient for imaging purposes. The majority of fluorescent cells obtained after a GFP-encoding DNA tattoo were keratinocytes (see Additional file 1).

**Figure 1:**
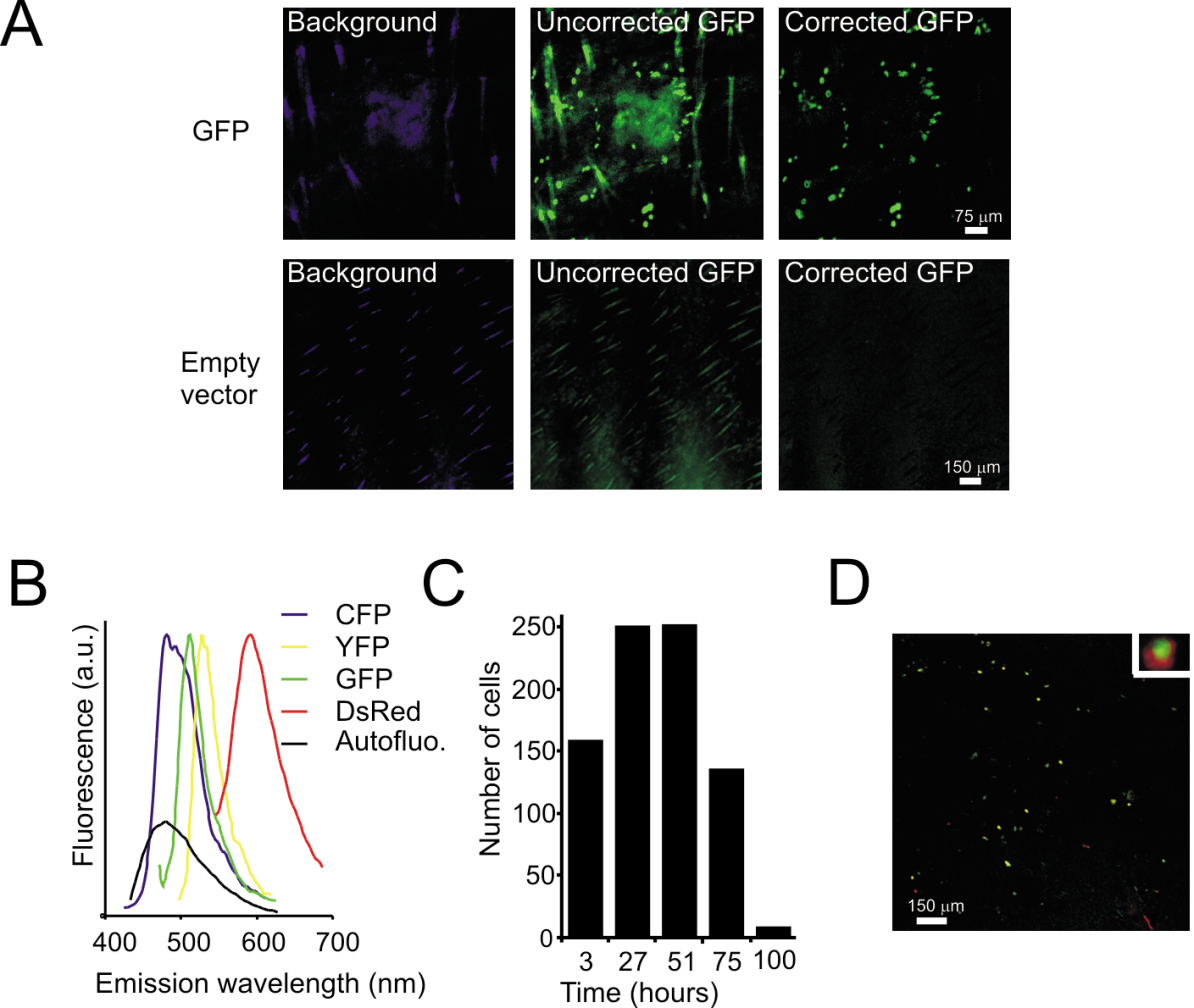
**Fluorescent protein expression in single cells after DNA tattooing on the skin of a living mouse**. A) Using a standard tattoo machine, a pVAX vector encoding GFP (upper panel) or an empty pVAX vector (lower panel) were tattooed into the skin of mice. Confocal GFP and autofluorescence images were collected as described in the text and GFP images were corrected for autofluorescence by subtracting 0.95× the autofluorescence image. B) The *in vivo *emission spectra of CFP, GFP, YFP or DsRed and autofluorescence, the latter at 405 nm excitation. C) The number of GFP expressing cells counted within the same 10 mm^2 ^skin area at different time points post tattoo, as indicated in the graph. D) Merged GFP and DsRed images of skin tattooed with a mixture of H2B-GFP- and DsRed-encoding vectors. Inset, a cell expressing both H2B-GFP and DsRed.

The uncorrected GFP images showed considerable autofluorescence of skin structures, in particular hair follicles, which was characterized by a substantially wider excitation and emission bandwidth. To discriminate the cells expressing GFP from these autofluorescent structures, autofluorescence was detected by imaging at 405 nm excitation and 410–440 nm emission. The resulting autofluorescence image was then subtracted from the GFP image, resulting in an autofluorescence-free GFP image (Figure 1A). Recent studies showed that in addition to GFP, multiple FPs can be simultaneously imaged *in vivo *[11-14]. In line with this, the DNA tattoo strategy also allowed (the simultaneous) detection of cells expressing a broad range of fluorescent reporters (FP), including cyan fluorescent protein (CFP), yellow fluorescent protein (YFP) and DsRed. As expected, the *in vivo *emission spectra of the tested FPs were similar to those reported *in vitro *(Figure 1B). When performing intravital imaging at several time points post-tattoo, the first fluorescent cells could be detected as early as 2 hours after DNA application. Although the number of transfected cells varies per tattoo (ranging from 100 to 300 cells per 10 mm^2^), the kinetics of expression are the same; within 24 hours the number of detectable cells increases approximately 2-fold and gradually declines over 4 days, at which point all expression has disappeared (this was observed for all tested FPs, and a representative GFP example is shown in Figure 1C). Adding to the flexibility of the DNA tattoo approach, tattoo application of a mixture of plasmids encoding different FPs (i.e. H2B-GFP and DsRed), resulted in detectable expression of both reporter genes in approximately a third of the transfected cells (Figure 1D). This number may be an underestimate because the wide range of observed intensities suggests that in many transfected cells one of the probes may be expressed below the level of detection.

**Figure 2:**
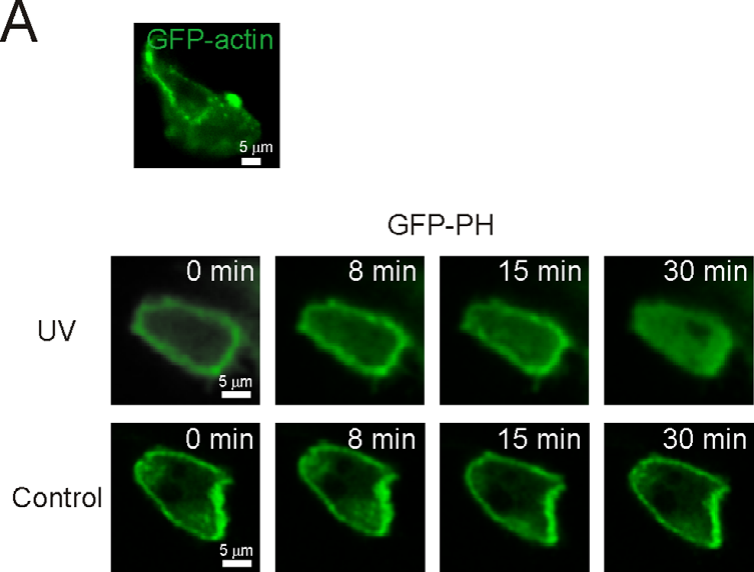
**Imaging of location-based biosensors with subcellular resolution**. A) Images of GFP-actin (top panel) and GFP-PH (lower panel) were collected from the medial section of cells using a confocal microscope. Note that GFP-PH translocated from the membrane to the cytosol in cells following UV-A exposure, but not in non-exposed (control) cells. The post-irradiation time is indicated in the images.

Having established the feasibility of monitoring biosensor expression in DNA tattoo-treated skin, we set out to determine the versatility of this approach to report on cellular processes and its ability to follow the fate of individual cells through time. Using a 63× 1.2NA water objective, captured skin images showed subcellular-resolution with details as small as ~300 nm (FWHM). Two GFP fusion proteins, GFP-actin and the PtdIns(4,5)*P*_2 _reporter GFP-PH, displayed their expected subcellular distribution: at the periphery of the cell in the cortical cytoskeleton and at the plasma membrane, respectively (Figure 2A). Importantly, we could reproduce the previously made *in vitro *observation that UV exposure of cells results in decreased PtdIns(4,5)*P*_2 _levels [15], as revealed by translocation of GFP-PH to the cytosol in cells irradiated with UV but not in control cells (Figure 2A, *lower panel*).

**Figure 3:**
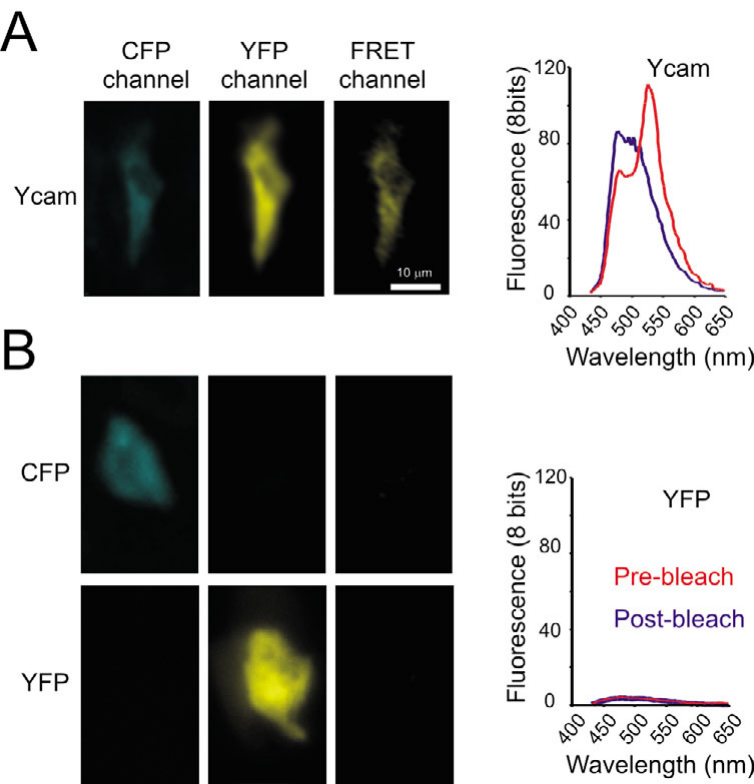
***In vivo *FRET**. A) CFP, YFP and sensitized emission (FRET) images (*Left panel*) and emission spectra (*Right panel*) were collected from skin cells expressing Ycam. The red and blue spectrum are pre- and post-YFP photo destruction respectively. Note the gain in CFP fluorescence after photo destruction of YFP. B) Control experiments for intravital FRET imaging. (*Left panel*) The collected FRET images in A were corrected as described in methods. FRET was not observed in cells expressing only CFP or YFP, consistent with proper correction. (*Right panel*) The emission spectrum acquired using CFP excitation wavelength (405 nm) of a cell expressing only YFP. The expression level of YFP is similar to that of Ycam in *A*. Note that YFP is inefficiently excited at 405 nm, and that photo destruction of YFP does not result in an increase of fluorescence at CFP wavelengths.

**Figure 4:**
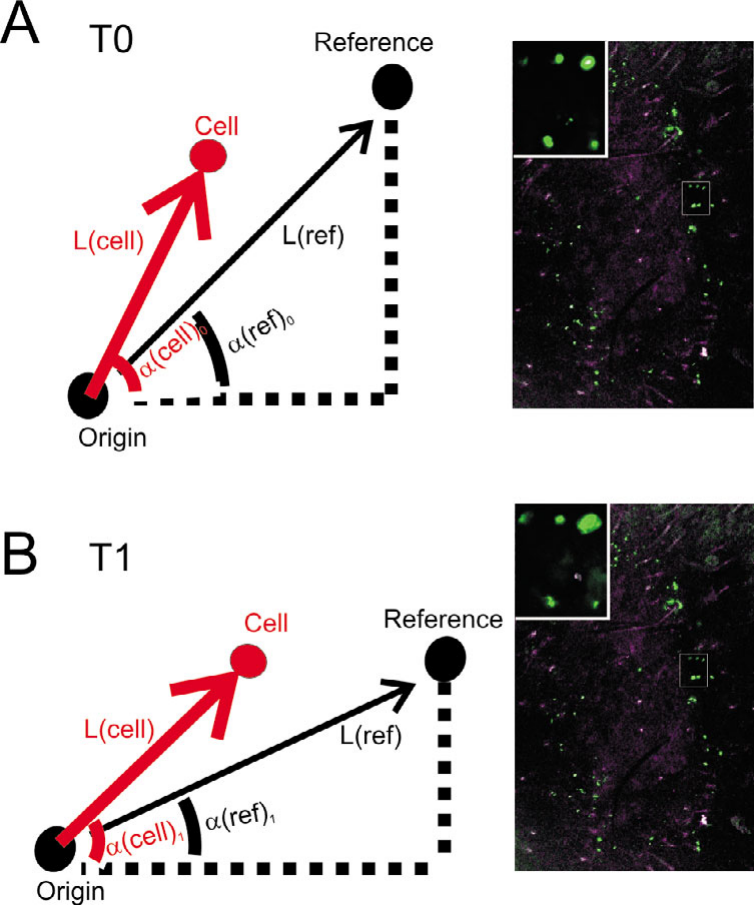
**Defined cells can be followed over multiple days**. A) In order to set up the coordinate system, two black ink reference points are tattooed on the abdomen of mice; origin and reference. At T0, the distance L(ref) and the angle a(ref) are measured between the two reference points. For cell coordinates, the distance L(cell) and the angle a(cell) are measured between the cell and origin. B) At a subsequent timepoint (T1), small rotations in the position of the mouse alter a(ref) and a(cell), but not L(ref) and L(cell). a(ref) is measured again, and a(cell) is corrected by the difference in a(ref) at T0 and T1. Knowing L(cell) and a(cell), the positions of the previously recorded cells can be re-calculated. (*Right panels*) Merge of GFP (green) and background (cyan) overview images of the skin of a mouse at T = 0 and T = 24 hours, imaged with a low-magnification objective (5×). The same cells are found back using the coordinate system. Insets represent the boxed regions, which have been imaged with a higher magnification objective (20×).

While many intracellular processes can be followed by monitoring the distribution of location-based probes, certain processes, such as conformational changes, are better revealed by (kinetic changes in) FRET. To assess the feasibility of *in vivo *FRET measurements and of the longitudinal follow-up of individual cells, we first applied the FRET-based Ca^2+ ^sensor 'Yellow chameleon' (Ycam) and its fluorescent constituents, CFP and YFP on separate skin patches of the same animal. In Ycam-tattooed skin areas FRET was readily detectable either by sensitized YFP emission (as described in [16], Figure 3A, *left panel*) or by acceptor photobleaching (Figure 3B, *right panel*, for control experiments, see Figure 3B). In order to follow individual cells for multiple days, we subsequently set up a coordinate system that allows one to relocate the same cell in subsequent imaging sessions. To this purpose, ink reference points are applied at the edge of the tattooed area and the position of cells of interest relative to these marker points is recorded during the first imaging session (Figure 4A). In subsequent imaging sessions, the coordinates of these cells combined with overview images at low (5×) magnification (Figure 4B) are then used to retrieve the position of defined cells (for details, see methods).

**Figure 5:**
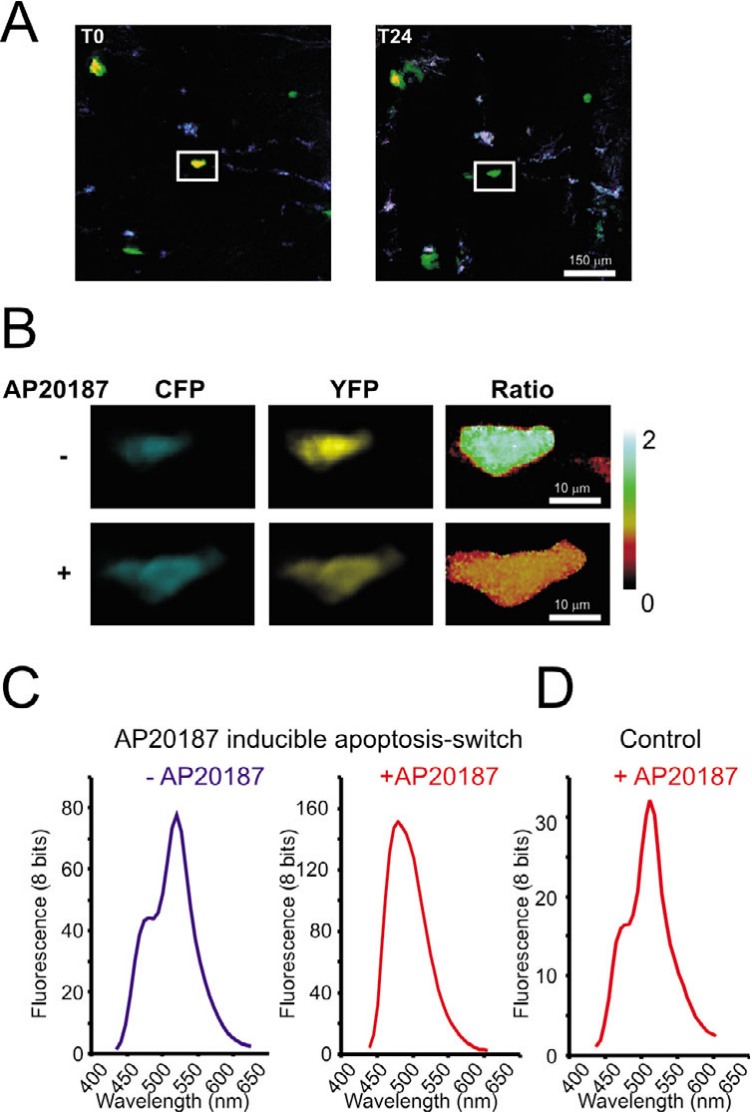
**Visualizing apoptosis in the skin of a living mouse by FRET**. A) An AP20187 sensitive 'apoptosis-switch' and FRET-based 'apoptosis-sensor' were tattooed on the skin of living mouse. Apoptosis was induced by injection of the chemical inducer of dimerization AP20187 in the peritoneal cavity of the mouse. Images were taken by confocal microscopy before and 24 hours after injection. Cells were identified by image registration at multiple time points as described in the text and supplementary data. A representative cell is boxed. Shown are merged images of the tdtomato, YFP and autofluorescence. B) CFP and YFP images of the cell as boxed in *A *imaged at higher resolution. Note the loss in FRET as a consequence of apoptosis induction. C) Emission spectrum of the cell boxed in *A *before injection of AP20187 (left, blue spectrum) and 24 hours after injection (right, red spectrum). D) Spectrum of a cell in the same animal as in C, double transfected with a control construct plus the FRET based apoptosis-sensor 24 hours after injection of AP20187.

To determine the potential of such longitudinal analysis, we performed a time-lapse experiment to study the involvement of caspase-3 in experimentally-induced apoptosis. To this purpose, a FRET-based sensor of caspase-3 activity, consisting of CFP and YFP moieties separated by a caspase-3 substrate site (modified from [17]) was utilized. DNA encoding this 'apoptosis-sensor' was mixed with DNA encoding an 'apoptosis-switch', consisting of a modified FK506- (tacrolimus) binding domain fused to caspase-9 [18], in a bicistronic configuration with tdtomato. This mixture was then applied to the abdominal skin of a mouse by DNA tattoo. In parallel, as a control for background apoptosis, a separate skin patch was tattooed with the apoptosis-sensor mixed with a vector encoding tdtomato only (pVax-tdtomato). To follow the consequences of apoptosis induction *in vivo*, autofluorescence, CFP and YFP images were recorded in the same set of cells expressing the apoptosis sensor, before and 24 hours after intraperitoneal injection of the suicide switch activator AP20187 (Figure 5A). Remarkably, after AP20187 administration the activation of caspase-3 was readily detected by a drop in YFP/CFP fluorescence ratio, as shown in the higher magnification images in Figure 5B. In line with this, after AP20187 administration, the clear peak of sensitized YFP emission disappeared (Figure 5C). Importantly, cells in skin areas of the same animal treated with the apoptosis-sensor plus control DNA did not show a change in emission spectrum upon AP20187 administration (Figure 5D). Collectively, the data from this experiment demonstrate that this technique allows one to induce a defined process in individual cells and to subsequently monitor the effects of this process (in this case caspase-3 activation) *in vivo *by an appropriate fluorescent sensor.

## Conclusion

Mouse transgenesis in combination with intravital imaging is time-consuming and can only be performed in specialized laboratories. Here we have presented a tattoo-based intravital imaging assay using techniques that are generally available. Clearly, this new approach that is based on the intradermal delivery of fluorescent reporters by DNA tattoo has its limitations. First, transgene expression obtained by the 'instant-transgenic' method as used here is confined to the epidermis and second, the expression of fluorescent reporters is restricted to a few days. In future studies, the duration of expression may be prolonged by enabling integration [19] or episomal maintenance of the plasmid used for transfection [20]. Furthermore, *in vivo *transfection using naked DNA can be achieved by various means in other tissues, including hepatocytes [21], myocytes [22] and lymphocytes [23]. More importantly, for many applications the abovementioned drawbacks are balanced by the substantial advantages provided by intravital DNA tattoo imaging. Firstly, mice expressing multiple cellular reporters can be generated in a time span of hours instead of months to years. Secondly, because it is possible to restrict expression of transgenes and reporters to a subset of cells amidst a population of unmodified cells, it is feasible to follow the behavior of gene-modified cells in isolation. In addition, this allows the retracing of individual cells in order to study these cells over a period of days. Thirdly, several tattoos with different combinations of transgenes and reporters can be compared within the same animal, thereby reducing experimental variation. Finally, imaging can be performed on widely available confocal or epifluorescent microscopes. These features suggest that intravital DNA tattoo imaging can form a highly rapid and versatile system to analyze cellular processes such as cell cycle progression, cellular differentiation or apoptosis in an *in vivo *setting.

## Methods

### DNA tattoo

The abdomen of the mouse was shaved and subsequently treated with depilation cream (Veet, Reckitt&Colmann, France). The nude abdominal surface was thoroughly rinsed to remove remaining cream, and a droplet of 20 μg DNA in 10 μl water was applied to the skin. Using a sterile disposable 11-needle bar (fine magnum 15, "challenge in colors" China) mounted on a rotary tattoo/permanent make-up device (Cold skin, B&A trading, Lijnden, the Netherlands) the DNA was tattooed in the skin over a surface of approximately 30 mm^2^[10]. During the 15 second tattoo, the needles oscillated at 100 Hz to a depth of 0.5 mm. Following application of the DNA, reference points were tattooed with black permanent make-up ink using a single point needle cartridge (Nouveau contour BV, Weert, Netherlands) mounted on a permanent make-up device (fine magnum 15, Aella, Medium-Tech, Berlin, Germany). All animal experiments were approved by the relevant institutional ethical committee (DEC) and performed in accordance with the local guidelines.

### Microscopy

Anesthetized mice (GFP-MHC class II mice [24] were a kind gift of R. Offringa) were transferred to a 37°C heated cage, mounted to an inverted TC-SP-AOBS confocal microscope (Leica, Mannheim, Germany). A 5× dry objective (0.15 HC PL Fluotar) and a 20× dry objective (0.7 HC) were used to collect overview images, and a 63× water objective (1.2 PL APO) to collect high resolution images.

### In vivo FRET imaging

*in vivo *FRET was measured by three independent methods; measurement of sensitized YFP emission, measurement of emission spectra, and measurement of the gain in CFP fluorescence upon YFP photo bleaching. For sensitized emission (YFP fluorescence upon CFP excitation), three images were collected: a CFP image excited at 405 nm and detected between 430 and 480 nm, a sensitized emission image excited at 405 nm and detected between 528 nm and 603 nm, and a YFP image excited at 514 nm and detected between 528 nm and 603 nm. Sensitized emission (FRET) was calculated from these images by correcting the sensitized emission image for leak-through of CFP fluorescence and fluorescence due to direct YFP excitation as described in [16]. Correction factors for leak-through of CFP and indirect excitation of YFP were determined on-line from cells expressing CFP or YFP only at different locations on the same animal. For every collected FRET image, images of cells expressing CFP or YFP only were collected subsequently to re-determine correction factors for laser fluctuations. Emission spectra were measured by collecting series of xyλ-images at 405 nm excitation and at 10 nm bandwidth emission starting at 430 nm and ending at 650 nm. No significant YFP emission was observed in emission spectra of cells expressing only YFP. Moreover, FRET observed by spectrum imaging was confirmed by the gain of CFP fluorescence after photo-destruction of YFP. The CFP gain was not a result of photo conversion of YFP during photo bleaching as described in [25], because YFP bleaching of skin cells expressing YFP only did not result in a significant increase in fluorescence at CFP wavelengths.

### Coordinate system to image the same cells for several days

Cells of interest were retraced using a coordinate system. The coordinate system is based on two black ink reference points, which are tattooed on the mouse abdomen: an origin and a reference. The distance L(ref) and the angle a(ref) between the origin and the reference are determined by a custom-made Visual Basic (v6.0) program by calling commands from the Leica macro tool package. For every cell coordinate, the distance L(cell) and the angle a(cell) between the cell and the origin are determined and saved. During the next imaging session, the re-anaesthetized animal is re-positioned on the confocal microscope. While L(ref) and L(cell) will be similar (but not identical, see below) at subsequent time-points, the reference a(ref) and all cell angles (a(cell)) will vary because of small rotations in the position of the mouse. To correct for this, a(ref) is re-determined, and all cell angles (a(cell)) are corrected for changes in a(ref). The positions of the cells are then recalculated from the corrected a(cell) values and L(cell). Stretching of the skin does result in small deviations in L in subsequent imaging sessions and therefore can result in misalignment of the position of cells up to 0.5 mm. To avoid this issue we use an overview image at low (5×) magnification to determine the exact coordinate of each cell (Figure 4). Once a single misalignment has been resolved, all positions (within a radius of 5 mm) are corrected for this misalignment.

## Authors' contributions

JvR and ADB conceived of and designed the study, performed the imaging, analysis and drafted the manuscript. KJ advised in FRET imaging and aided in drafting the manuscript. JRH, ND were involved in the experiments resulting in Figure 2A lower panel, DMS provided materials, JBAGH participated in the coordination. TNMS participated in the study design and coordination and helped to draft the manuscript.

## Supplementary Material

Additional File 1 **The fluorescent cells observed after DNA tattooing are predominately keratinocytes**. This section describes that the fluorescent cells observed after DNA tattooing are predominately keratinocytes. Click here for file
