# Supplementary material for: Intravital imaging of fluorescent markers and FRET probes by DNA tattooing
Source: BMC Biotechnol. 2007 Jan 3;7:2. doi: 10.1186/1472-6750-7-2 (PMC1779781; doi:10.1186/1472-6750-7-2)
Supplement: Additional File 1 — The fluorescent cells observed after DNA tattooing are predominately keratinocytes. This section describes that the fluorescent cells observed after DNA tattooing are predominately keratinocytes. [file 1472-6750-7-2-S1.pdf]

The fluorescent cells observed after DNA tattooing are predominately keratinocytes

The epidermal layer of mouse skin is nearly exclusively composed of keratinocytes and antigen presenting cells (Langerhans cells). Judged by morphology, a large fraction of the cells expressing FPs after DNA tattooing are keratinocytes. In line with this, application of a GFP-encoding plasmid in which transcription is driven by the keratinocyte-specific K14 promoter yields detectable expression in a high number of cells (approximately 50% as compared to the number of FP-positive cells observed upon introduction of a CMV IE-driven transgene) (**additional file Figure 1A)**. Because the K14 promoter is 10-100 fold weaker than CMV IE-promoter, the number of GFP-expressing keratinocytes observed upon application of the K14-driven GFP vector is likely to be an underestimate. To determine whether transgene-expression can also be observed in skin-resident antigen-presenting cells, we tattooed an H2B-mRFP-encoding plasmid to transgenic mice in which all Langerhans cells are marked by GFP expression (GFP fusion to MHC class II [24]). While both GFP-marked Langerhans and mRFP-expressing cells are readily observed, co-expression of the two FPs is rare (**additional file Figure 1B**), indicating that only small numbers of Langerhans cells are transfected after DNA tattooing. In conclusion, although both keratinocytes and APCs are present in the epidermal layer of the skin, the fluorescent epidermal cells that are observed after DNA tattooing are predominately keratinocytes.

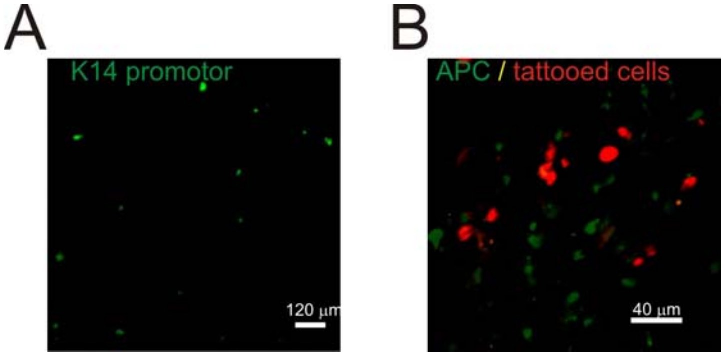

Additional material Figure 1

DNA tattooing results in fluorescent keratinocytes. A) GFP expression observed upon application of a keratinocyte-specific K14 promoter-driven GFP transgene. Application of DNA encoding K14 or CMV IE-driven GFP transgenes resulted in approximately 8 or 16 fluorescent cells/mm^2 respectively. B) Transgenic mice in which all Langerhans cells express GFP were tattooed with a plasmid encoding a CMV IE-driven H2B-mRFP transgene. Depicted is a merge of a GFP and mRFP image.
